# Cytokine TNF-α promotes invasion and metastasis of gastric cancer by down-regulating Pentraxin3

**DOI:** 10.7150/jca.39562

**Published:** 2020-01-17

**Authors:** Xinye Cui, Han Zhang, An'na Cao, Liang Cao, Xiang Hu

**Affiliations:** 1 Department of General Surgery, The First Affiliated Hospital, Dalian Medical University, Dalian 116011,China; 2 Department of Pathology, Dalian Medical University, Dalian 116044, People's Republic of China

**Keywords:** PTX3, Milky spot, TNF-α, Gastric cancer, EMT

## Abstract

As a novel multifaceted player in cancer, Pentraxin3(PTX3) was recognized to be a possible factor related with tumor development. Recent researches have indicated that PTX3 is involved in immune response, inflammation, as well as cancer, and is greatly controlled by numerous cytokines. Tumor necrosis factor (TNF-α) is an imperative cytokine that demonstrates an extensive array of biological consequences in gastric cancer advancement. Here, we inspected the expression of PTX3 in gastric carcinoma tissues along with gastric cell lines and established that PTX3 was suggestively inferior in gastric cancer tissue and cells. The treatment of the gastric cell lines BGC-823 as well as SGC-7901 with rhTNF-α caused substantial decrease in the expression of PTX3. Furthermore, PTX3 controlled the capability of cell migration, invasion as well as epithelial-mesenchymal transition (EMT) in gastric cancer cell lines mediated by TNF-α. Additionally, PTX3 upregulation inhibited tumorigenicity *in vivo* and could be reversed by exogenous TNF-α. Conversely, overexpression of PTX3 inhibited progress both *in vitro* as well as *in vivo* in gastric cancer mediated by TNF-α. Further studies are necessary to demonstrate the mechanism of interaction between PTX3 and cytokines.

## Introduction

Gastric cancer is the fourth utmost commonly occurring cancer and the second foremost reason of cancer-associated death 1. Peritoneal diffusion often occurs in the metastasis of gastric cancer, which is the most severe issue affecting the long-term survival of patients. Peritoneal metastasis is a common pattern of distant metastasis of gastric cancer, as the milky spot is the main malignant cell implantation site in peritoneal transmission; the study of the mechanism is mainly focused on milky spot 2. Milky spot is composed of a great number of macrophages as well as lymphocytes, which can promote cancer cells colonization, survival and metastasis by interaction with tumor cells through the secretion of chemotactic factor 3,4,5. Tumor necrosis factor (TNF-α) is a member of cytokine superfamily and is convoluted in the preservation and dynamic balance of immune system, inflammation as well as host defense. However, TNF-α is thought to be incorporated in chronic inflammation, autoimmune as well as malignant diseases. At the same time, inflammatory cytokine TNF-α has been reported to have vital roles in tumor progress as well as metastasis.

PTX3 (also referred as TSG-14) is the prototypic member of the long-pentraxin subfamily 6. Initially, PTX3 was a pattern recognition receptor, which participated in inflammatory response as well as pathogen identification related functions 7. Then, different studies have shown that PTX3 plays a variety of roles in wound healing, tissue restoration, cardiovascular disease as well as infectious diseases. The expression of PTX3 is considered to be related to inflammation and a biomarker of the above pathological state. In addition, PTX3 has an involvement in various phases of tumor development, comprising tumorigenesis, angiogenesis, metastasis, and tumor immunomodulation 8. As the tumor cells are influenced by PTX3, it is very important to elucidate the effects of PTX3 on the above-mentioned aspects *in vitro* as well as *in vivo*. Currently, no reports regarding the expression of PTX3 in gastric cancer and its paracancerous tissues have been published. In addition, although the mechanism of action of TNF-α in tumors has been widely proven during recent years, studies have not mentioned the interaction between TNF-α and PTX3 in gastric cancer metastasis and EMT. We infer the correlation between PTX3 and TNF-α during the progress of gastric cancer cells, which provides a foundation of TNF-α could regulate PTX3 in a negative way* in vitro* as well as *in vivo*. Overexpression of PTX3 might prevent the cell incursion plus EMT in cancer cells *in vitro* as well as *in vivo*. We infer the link between PTX3 and TNF-α, which provides a foundation for additional studies on the mechanism of interaction between PTX3 and macrophage related factors in milky spot. We hypothesize that PTX3 might serve as a new target for gastric cancer treatment.

## Materials and Methods

### Bioinformatics analysis

Gene data of gastric cancer samples were acquired from the TCGA data portal. PTX3 mRNA manifestation levels in gastric cancer samples were analyzed using GraphPad Prism software.

### Tissue collection

50 recently frozen tissue samples and analogous normal tissue samples from near the tumor specimens (≥5 cm) collection site were obtained from gastric cancer patients (20 males; 30 females; 44-71 years; mean 56), who underwent curative resection for primary gastric adenocarcinoma (or gastric carcinoma) at the First Affiliated Hospital of Dalian Medical University. These patients provided informed consent to allow their tissue samples to be used for experimentation. The First Affiliated Hospital Institutional Review Board provided approval for this process. Each tissue sample was separated into two parts for quantitative real-time PCR (qRT-PCR) as well as western blot.

### Cell culture and animals

Human normal gastric epithelial cells (GES-1) along with gastric cancer cell lines (BGC-823, SGC-7901) were acquired from the Laboratory of Pathology at Dalian, Medical University. The GES-1 and BGC-823 cells were cultured in DMEM (Gibco, USA) with 10% FBS. The SGC-7901 cells was cultured in RPMI-1640 medium (Gibco, USA) complemented with 10% FBS. Incubations was done at 37 °C with a 5% CO_2_ atmosphere.

BALB/c nude mice (age 6-8 weeks) were bought from Vital River Laboratory of Animal Technology (Beijing, China) and were raised following guidelines of the Animal Care and Use Committee of Dalian Medical University.

In order to explain the influence of exogenous TNF-α on the regulation of PTX3, we implemented assays on samples that had been subjected to various dosages of rhTNF-α.

### qRT-PCR

Total RNA was obtained from cultured cells using Trizol (Transgen, China). 1μg of the total RNA was reverse-transcribed using the SuperMiX kit (Transgen, China). The mRNA manifestation level of PTX3 was determined using real-time PCR. The iCycler™ Real Time System as well as a SYBR Premix EX Tag Master mixture kit (Transgen, China) were utilized for this examination.

### Plasmids, lentivirus, antibodies and reagents

PTX3 expressing plasmid was produced by cloning PTX3 cDNA into a pcDNA3.1 vector obtained from GenePharma Company (Suzhou, China), and the process was conducted on a Lipofectamine3000 system. BGC-823 and SGC-7901 cells were transfected with PTX3 or a negative control (NC) plasmid. Lentivirus vectors comprising LV5-PTX3 overexpressing PTX3(PTX3) and the negative control (LV5-NC) were bought from GenePharma Company (Suzhou, China). The transfected cells were treated with puromycin (Clontech, USA). Anti-PTX3, anti-E-cadherin and all other antibodies and recombinant human TNF-α were acquired from Proteintech Group, Inc. (Wuhan, China), while the secondary antibodies were bought from Santa Cruz Biotechnology (USA).

### Migration and invasion assay

Equal quantities of BGC-823 and SGC-7901 cells were cultured in MEM Alpha Modification medium for 24 hours. For the transwell migration assay, 1 × 10^5^ cells were sowed without serum into Transwell chambers (Corning, USA). The medium complemented with 20% serum and was mixed into the bottom chamber. 12-16 hours later, the upper cells were cleaned using cotton swabs. 1% crystal violet was utilized for staining the cells and staining in an arbitrarily selected area was analyzed using an optical microscope. For the invasion assay, 1 × 10^5^ cells were sowed without serum into Transwell chambers (Corning, USA) and pretreated with Matrigel (BD, USA). The medium complemented with 20% serum was mixed into the bottom chamber. 12-16 hours later, the upper cells were cleaned using cotton swabs. 1% crystal violet was utilized to stain the cells and staining in an arbitrarily selected area was analyzed using an optical microscope.

### Western blotting

After washing with PBS, the transfected cells were treated with a whole cell lysis assay kit (KeyGEN BioTECH, China). Protein concentrations were assessed using a Easy II Protein Quantitative Kit (Transgen, China). Equal quantities of protein were separated on 10% SDS-PAGE and then transmitted to PVDF membranes (Millipore, USA). Following blocking with 5% non-fat milk, the blots were incubated with the relevant antibodies overnight at 4 °C. Finally, the transfer membranes were incubated with secondary antibodies and images of the bands were captured using an ODYSSEY infrared imaging system.

### Mouse xenograft model

Animal experiments were conducted following Guidelines for the Care and Use of Laboratory Animals (NIH). Twenty BALB/C nude mice were randomly separated into four groups. The negative control group (LV5-NC) and LV5-PTX3-transfected BGC-823 cells (3 × 10^6^) in 100μl PBS were subcutaneously inoculated into the left flank of the animals. In order to evaluate the effectiveness of TNF-αon PTX3 in tumorigenicity, the animals were randomly treated with Rh-TNF-α (2μg/kg) or 0.9% saline for three days. Animals were treated again with their allocated treatment once every 2 days. The tumor sizes were quantified by means of a Vernier caliper and documented on alternate days. Tumor volume was calculated by following formula: tumor volume [mm]^3^ = (length [mm]) (width [mm])^2^ × 0.5. Following the injection for 25 days, the xenograft tumors were removed from the animals and investigated by immunohistochemistry.

### Immunohistochemical analysis

Tumor tissues were acquired from mouse xenograft models. Paraffin-embedded tissues were sliced into 5μm thick portions and were secured onto glass slides and stained with their respective antibodies. Following deparaffinization in xylene and rehydration with ethanol, they were preincubated with 10% normal goat serum and then incubated with primary antibodies overnight at 4°C. Next, the slides were washed with PBS, incubated with the secondary antibody at 37°C for 10 min and washed again. Thereafter, they were incubated with peroxidase conjugated-biotin streptavidin complex for 10 min. Finally, the slides were analyzed using 3,3′-diaminobenzidine plus hematoxylin. Scoring was done by multiplying the intensity (0-3) with the extent (0-100) of staining.

### Statistical analysis

Analysis was done with SPSS version 11.0. Data are presented as mean ± SD. Student's t-test and one-way ANOVA were performed. A P value of < 0.05 was considered to signify statistical significance. GraphPad Prism software was utilized to analyze the clinical data.

## Results

### Expression of PTX3 in human gastric cancer cells as well as tissues

PTX3 is overexpressed in soft tissue sarcomas 9, lung cancer 10,11 myeloproliferative neoplasms 12, pancreatic carcinoma 13, gliomas 14, and hepatocellular carcinoma 15. But in other aspect, Margheri et al. 16 established that PTX3 overexpression decreased the angiogenic activity *in vitro* as well as *in vivo* due to its FGF2-neutralizing capability. Bonavitae et al. evaluated its function in carcinogenesis and detected that PTX3 deficiency was related with chemically prompted mesenchymal and susceptibility to epithelial carcinogenesis 17, suggesting that PTX3 has an inhibitory effect on tumor.

**Figure 1:**
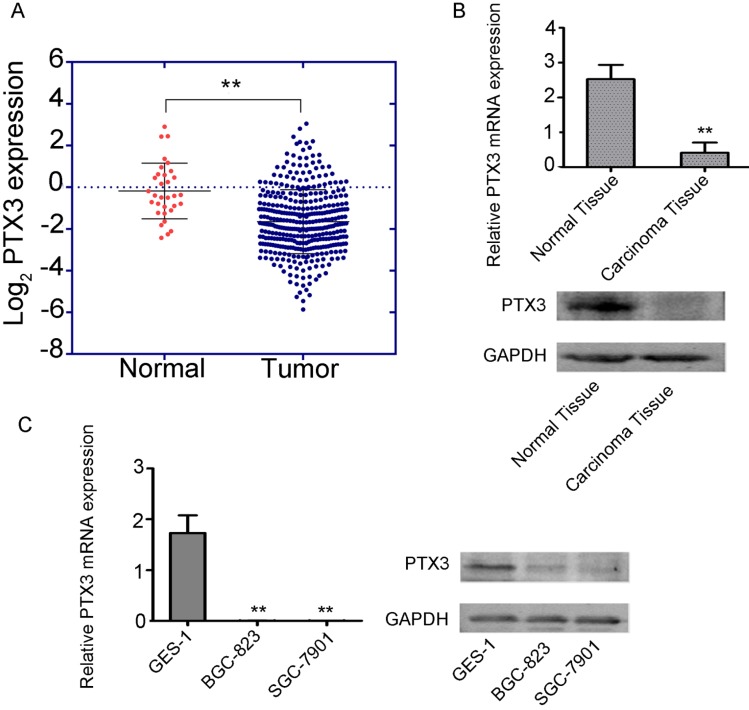
Low expression of PTX3 in human gastric cancer.** A.** Cancer with PTX3 transcripts evidently had lower manifestation (n = 375) compare to normal gastric tissues (n = 32) from the TCGA database (** P < 0.01). **B.** PTX3 expression levels were detected in the gastric cancer tissue as well as the adjoining normal tissue samples via qRT-PCR and Western blotting (**P<0.01). **C.** PTX3 manifestation levels were identified in human normal gastric epithelial cells (GES-1) and human gastric cancer cells (BGC-823 and SGC-7901) via qRT-PCR and western blotting (**P < 0.01).

To examine the function of PTX3 in tumorigenesis, we inspected the gastric cancer database of TCGA to assess the differential manifestation of PTX3, which specified that cancer with PTX3 transcripts (n = 375) had suggestively lower manifestation level compare to normal samples (n =32) (Fig. 1A). Additionally, we compared the manifestation of PTX3 between the carcinoma tissue and paracancerous tissue in 50 patients, which showed that PTX3 was less expressed in gastric cancer tissues, and greatly expressed in normal tissues(Fig. 1B). Moreover, to authenticate the manifestation of PTX3 gene in gastric cancer, we used BGC-823, SGC-7901 as well as GES-1 cells, which got the same results (Fig. 1C). In brief, these outcomes recommend that PTX3 is unusually less expressed in human gastric cancer patients as well as cell lines.

### The effect of macrophage factor TNF-α on the expression of PTX3 *in vitro*

TNF-α is considered to participate in the process of pathology like chronic inflammation, autoimmunity as well as malignant disease. In addition, many factors could regulate the expression of PTX3. For instance, inflammatory cytokines (IL-1β, TNFα), microbial components (LPS, lipoarabinomannans), TLR agonists. Otherwise, it has been documented that in the model of epithelial carcinogenesis, cytokines TNF-α, was suggestively greater in PTX3^-^/^-^ tumor homogenates compare to PTX3^+^/^+^ 17. Therefore, we thought there might be a possible link between TNF-α and PTX3 in the gastric cells. We inspected the impacts of rhTNF-α on PTX3 in the gastric cancer cells.

**Figure 2:**
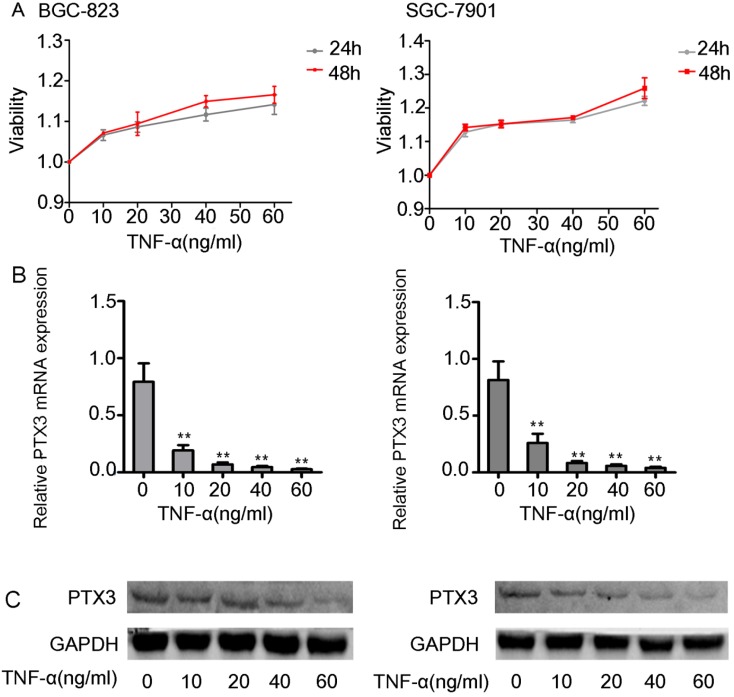
Effects of exogenous TNF-α on the expression of the PTX3 in gastric cancer cells.** A.** The viability assay of rhTNF-α on the BGC-823 as well as SGC-7901 cells through the CCK-8 assay.There had no inhibitory and toxic effects on the two cells at 24 hours and 48 hours at different concentrations of rhTNF-α (0,10,20,40,60ng/ml). **B.**Five different concentrations (0,10,20,40,60ng/ml) of rhTNF-α were added to BGC-823 as well as SGC-7901 cells. Both cells displayed suggestively reduced expression of PTX3 in a concentration-dependent manner (0,10,20,40,60ng/ml) via qRT-PCR and western blotting.

First we did the viability assay of rhTNF-α on the BGC-823 as well as SGC-7901 cells. We established that there had no inhibitory and toxic effects on the two cells at 24 hours and 48 hours at different concentrations of rhTNF-α (0, 10, 20, 40, 60ng/ml) (Fig. 2A). Next, we treated both cells in different concentrations of rhTNF-α as mentioned above and it demonstrated significant decrease in expression of PTX3 in a concentration-dependent manner (0, 10, 20, 40, 60 ng/ml) in qRT-PCR (24h) and western blotting (48h) (Fig. 2B,C). Collectively, these results suggested that TNF-α could regulate PTX3 in a negative way in both the BGC-823 as well as SGC-7901 cells.

### Abnormally expressed PTX3 participates in gastric carcinoma progression mediated by TNF-α

**Figure 3:**
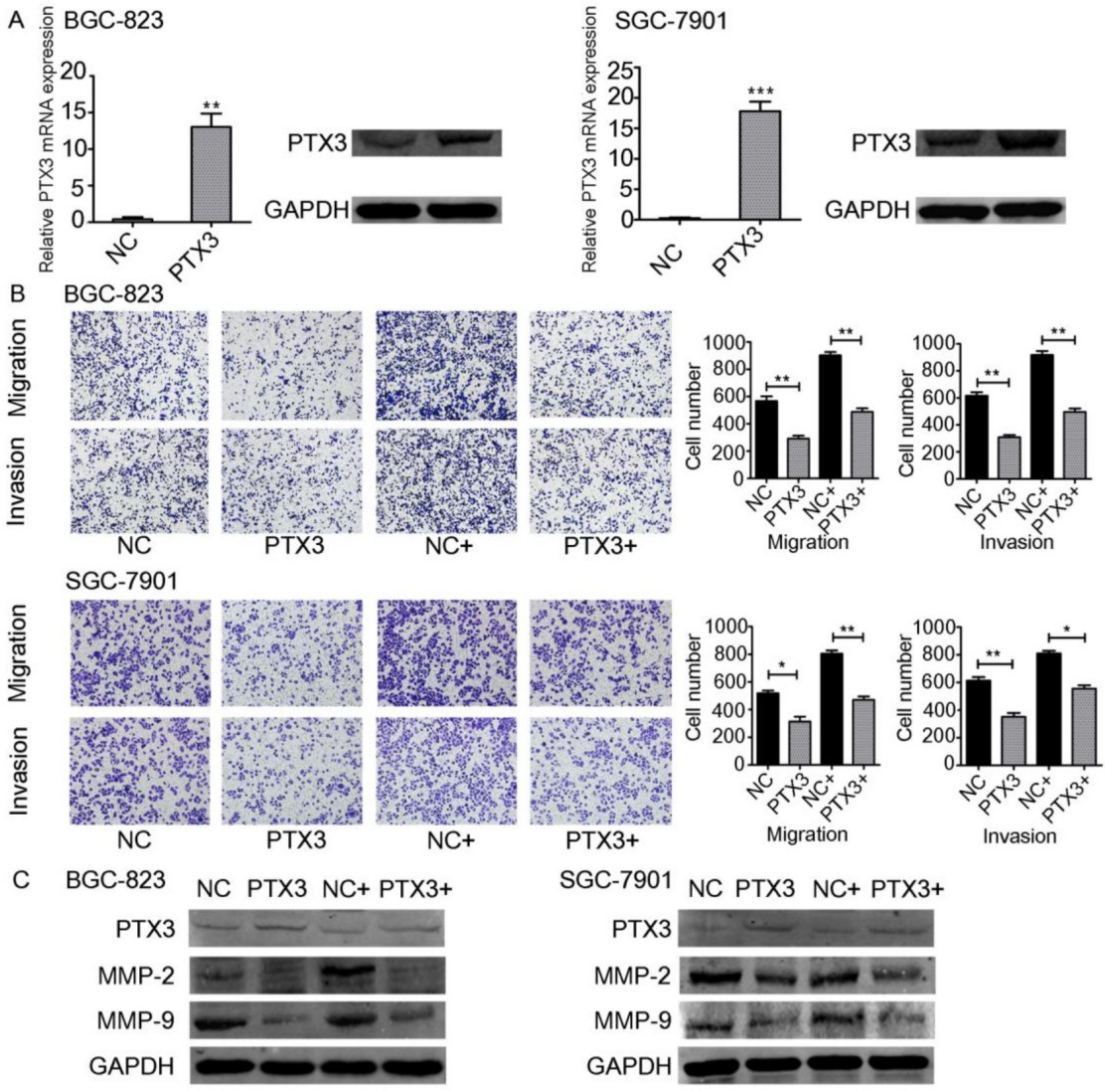
Effect of TNF-α on PTX3 in the migration as well as invasion of gastric cancer cell lines.** A.** BGC-823 as well as SGC-7901 cells were transfected with PTX3 upregulation or a negative control (NC) plasmid. Western blotting as well as qRT-PCR of PTX3 upregulation efficiency. **B.** Representative images from cell migration and invasion assay after treated by rhTNF-α(20ng/ml) in PTX3 upregulation cells and NC cells. **C.** Western blotting investigation of PTX3, MMP-2, MMP-9 in PTX3 upregulation cells and NC cells after treated with rhTNF-α(20ng/ml) for 48h.

TNF-α upregulates the emission of matrix metalloproteinase-9 (MMP-9) from tumor cells 18. Moreover, some studies have indicated the relevance between the inflammation and cancer progression. In particular, TNF-α has been documented to participate in the instigation as well as advancement of cancer. TNF-α is a multifunctional cytokine that modulates various phases of cancer cell phenotypes, for example cell propagation, migration, invasion, as well as instigating the death of tumor cells. TNF-α has also been documented to stimulate so-called epithelial-mesenchymal transition (EMT), which includes changes in both morphological and invasive phenotypes 19, 20. On the other hand, it has been documented that PTX3 participated in the process of migration as well as invasion of the cancer cells 21, 22. Since PTX3 can be regulated by TNF-α, we believed that PTX3 contributes in gastric carcinoma advancement facilitated by exogenous TNF-α. To investigate this question, we used plasmids to overexpress PTX3 in BGC-823 as well as SGC-7901 cells. PTX3 mRNA manifestation levels were significantly increased in both cancer cells compared to the control group (NC).Furthermore, PTX3 protein levels were also raised suggestively (Fig. 3A).

In order to explain the role of TNF-α on PTX3 in the migration as well as invasion of gastric cancer cells, we performed transwell assays. Overexpression of PTX3 obviously repressed cell migration and invasion in BGC-823 as well as SGC-7901 cells. In addition, when we used rhTNF-α to treat both cells, it abolished this inhibition in TNF-α-treated overexpressed PTX3 cells (Fig. 3B,C). We established that upregulation of PTX3 noticeably reduced the gastric carcinoma migration and invasion capacity mediated by TNF-α.

### Overexpression of PTX3 inhibit the EMT process of gastric cancer cells mediated by TNF-α

**Figure 4:**
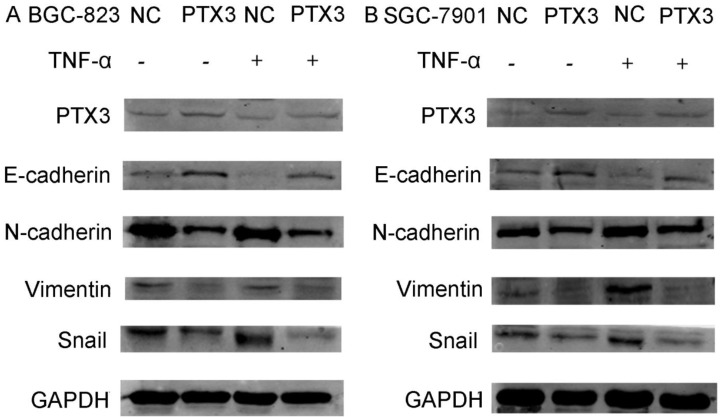
Protein levels of PTX3, E-cadherin, N-cadherin, Vimentin and Snail as detected by western blot in gastric cancer cells after the NC group or the PTX3 overexpression group was treated with rhTNF-α(20ng/ml) for 48 h. GAPDH served as a loading control.

Numerous previous studies have confirmed the importance of the activation of the EMT process for tumor development, invasion and metastasis. To identify the mechanism of PTX3 on the process of EMT of gastric cancer cells mediated by TNF-α, we examined typical EMT markers by western blot. As presented in Fig.4, overexpression of PTX3 suggestively reduced Snail1, N-cadherin as well as Vimentin manifestation, but augmented E-cadherin manifestation levels. However, when we used rhTNF- α to treat cells, it abolished this inhibition in TNF-α- treated overexpressed PTX3 cells. These results indicated that upregulation of PTX3 inhibit the EMT procedure of gastric cancer cells mediated by TNF-α.

### Overexpression of PTX3 inhibits tumorigenicity mediated by TNF-α

**Figure 5:**
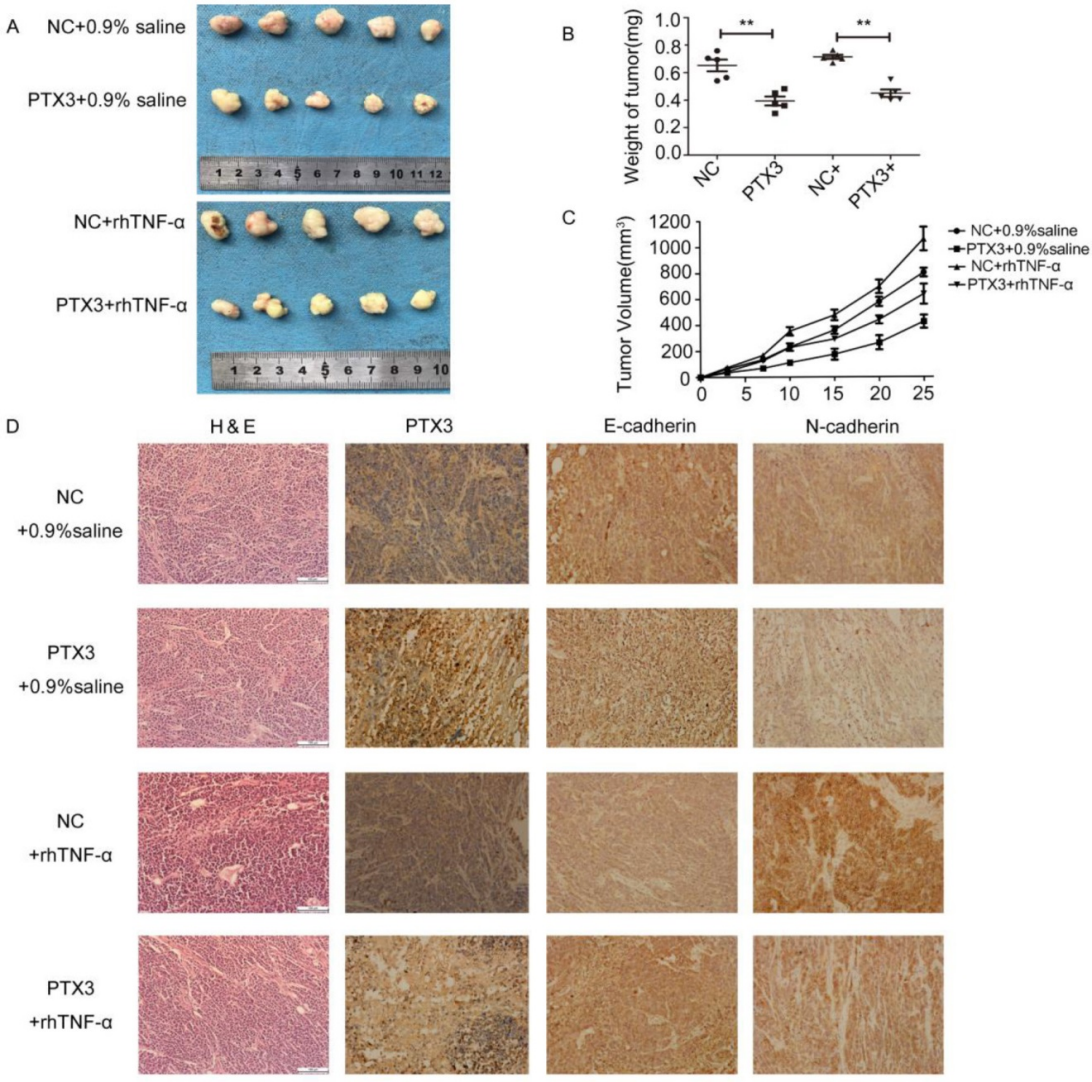
TNF-αreversed the inhibitory effect of tumorigenicity mediated by upregulation PTX3 on EMT *in vivo*. **A.** Descriptions of tumor xenografts and **B** tumor weight for tumor xenografts treated with 0.9% saline or rhTNF-α. **P < 0.01. **C.** Tumor growth curves were sketched up to day 25. **D.** Immunohistochemical detection of PTX3, E-cadherin, N-cadherin in tumor xenografts.

To inspect the anti-tumor effects of PTX3 upregulation on tumor development mediated by TNF-α *in vivo*, we developed xenograft tumor model by subcutaneously inoculating gastric cancer BGC-823 cells that had been transfected with lentivirus vectors including LV5-PTX3 overexpress PTX3 and the negative control (LV5-NC). Compared to the LV5-NC control group, the volume as well as weight of LV5-PTX3 group augmented at a lower rate. The typical tumor volume as well as weight of TNF-α- treated LV5-PTX3 tumors was distinctly lesser than that of TNF-α-treated LV5-NC tumors (Fig.5A,B,C). In addition, we established that higher manifestation level of PTX3 apparently lessened the manifestation of E-cadherin as well as N-cadherin in the tumor tissues (Fig.5D). Likewise, tissues produced from TNF-α- treated LV5-NC cells indicated the highest manifestation levels of E-cadherin and N-cadherin and lowest expression levels of PTX3 than other tissues. These outcomes indicate that overexpression of PTX3 inhibits tumorigenicity mediated by TNF-α.

## Discussion

PTX3 is manufactured by innate immune cells. It intermingles with multiple ligands and has an important function in innate immunity, inflammatory regulation as well as matrix deposition 23. PTX3 seems to have a dual function in cancer. Its overexpression is considered to be an adverse prognostic indicator in some cases, but in others, it is considered to be antitumor because of its anti-angiogenic as well as anti-tumor properties. The double-sided effect of PTX3 recommends that its function in cancer may be precisely reliant on the type of cancer, cell origin and tumor microenvironment. Here, we have demonstrated low manifestation of PTX3 in gastric cancer. Supporting the results, TCGA database, clinical gastric tissue and gastric cells were used for analysis. Therefore, PTX3 levels might possibly act as a precise anti-oncogene incorporated in gastric carcinoma advancement.

Tumor cells, tumor associated fibroblasts, endothelial cells as well as infiltrating immune cells 24, 25 create autocrine plus paracrine loops of communication and crosstalk that severely influence and occasionally reprogram cancer fate. It is of great significance to study the link between macrophage and cancer-related molecules in invasion as well as metastasis of gastric cancer. TNF-α has been reported to play crucial roles in cancer progression and metastasis 26,18. Moreover, it has been shown that in the model of epithelial carcinogenesis, TNF-α was suggestively greater in PTX3-/- tumor homogenates compare to PTX3+/+ 17. Nevertheless, there was no proof to recognize the link of the PTX3 with TNF-α in gastric cancer. In order to inspect the link between PTX3 and TNF-α in gastric cancer, we established that exogenous TNF-α might directly influence the manifestation of the PTX3 in the BGC-823 as well as SGC-7901 cells. Additionally, overexpression of PTX3 might decrease the gastric carcinoma migration as well as invasion capacity mediated by TNF-α according to our data. However, the mechanisms on how PTX3 expression is regulated by TNF-α still requires further studies.

Because the milky spot is composed of a large number of macrophages and lymphocytes, which can promote cancer cell colonization, survival and metastasis by the interaction with tumor cells through the secretion of chemotactic factor, it is important to study the role of macrophage and cancer related molecules in metastasis. The process of EMT shows epithelial cancer cells dissemination, which includes motility, invasiveness plus anti-apoptosis, and results in the distribution of cells and settlement in distant tissues 27,28. We revealed that overexpression of PTX3 treatment augmented the manifestation of E-cadherin but reduced the manifestation of Snail1, N-cadherin and Vimentin (Fig.4,5). On the other side, when we added rhTNF-α, which could reverse the inhibitory effect of upregulation of PTX3 on EMT *in vitro* as well as *in vivo*.

## Conclusion

Our study demonstrates the low expression of PTX3 in gastric samples and gastric cancer cell lines. TNF-α can downregulate PTX3 expression. Furthermore, upregulation of PTX3 may hamper cell migration, invasion, and EMT through the mediation of TNF-α. These results indicate the potential connection between PTX3 and cytokines, indicating an investigational basis for its usage as a tumor biomarker and expanding its possible clinical importance.
